# Length of Stay Increases 90-day Readmission Rates in Patients Undergoing Primary Total Joint Arthroplasty

**DOI:** 10.5435/JAAOSGlobal-D-21-00271

**Published:** 2022-03-16

**Authors:** Jorge Benito, Justin Stafford, Hyrum Judd, Mitchell Ng, Arturo Corces, Martin W. Roche

**Affiliations:** 1 From the Larkin Medical Center, Department of Orthopedic Surgery, Miami, FL (Dr. Benito, Dr. Stafford, Dr. Judd, and Dr. Corces), and the Holy Cross Hospital, Orthopedic Research Institute, Ft. Lauderdale, FL (Dr. Ng, and Dr. Roche).

## Abstract

Postoperative length of stay (LOS) of 1 day compared with that of 2 to 4 days after primary total knee arthroplasty (TKA) or total hip arthroplasty (THA) and 90-day readmissions.

**Methods:**

PearlDiver identified TKA (n = 648,758) and THA patients (n = 346,732) between 2005 and 2014. Groups consisted of control (LOS = 1 day) and study (LOS = 2 to 4 days) groups. Study and control groups were matched to age, sex, and Elixhauser Comorbidity Index. Logistic regression analysis and odds ratio analyzed 90-day readmission rates. *P* < 0.05 was statistically significant.

**Results:**

TKA patients' LOS of 2 days (odds ratio [OR], 2.89; 95% confidence interval [CI], 2.77 to 3.00), LOS of 3 days (OR, 2.80; 95% CI, 2.69 to 2.91), and LOS of 4 days (OR, 2.83; 95% CI, 2.72 to 2.95) had greater 90-day readmission compared with LOS of 1 day (*P* < 0.05). THA patients with an LOS of 2 days (OR, 2.93; 95% CI, 2.77 to 3.10), an LOS of 3 days (OR, 2.91; 95% CI, 2.75 to 3.07), or an LOS of 4 days (OR, 2.91; 95% CI, 2.73 to 3.05) had greater 90-day readmission compared with an LOS of 1 day (*P* < 0.05).

**Conclusion:**

LOS >1 day has greater odds of 90-day readmission after an index procedure. Efficient progression to early discharge regarding patient-specific risk factors plays a large role in preventing readmission.

Recent efforts within total joint arthroplasty (TJA) have focused on reducing the episode of care costs without sacrificing quality of care and patient outcomes.^1-3^ Minimizing postoperative readmission rates decreases overall costs and healthcare burden, as well as postprocedural morbidity, and has become a major surrogate for assessing surgical outcomes.^1-3^ However, in efforts to identify those specific risk factors which may predispose to unplanned readmission, studies present conflicting evidence, particularly regarding the significance of in-hospital length of stay (LOS) after surgery.

Mean hospital LOS decreased from 9.1 days to 3.7 days, whereas 30-day all-cause readmission rates increased from 5.9% to 8.5%; however, the number of comorbid illnesses per patient also increased over that period.^4^ The authors proposed that decreasing LOS seemed to be associated with the rising incidence of unplanned readmission in TJA.

Recent studies have attempted to demonstrate an association between decreasing LOS and increased readmission rates, but in fact, most authors show that early discharge to home with a well-established perioperative and rehabilitation protocol may actually have a protective effect on unplanned readmission.^5,6^ Although most studies have used 30-day readmission rates as an end point, recent studies support equivalent 90-day readmission rates among patients with shorter LOS.^5,6^ To the best of our knowledge, however, no study has examined 90-day readmission rates in both total knee arthroplasty (TKA) and total hip arthroplasty (THA) patients using a large administrative database, as stratified specifically by day of discharge. In addition, studies focused primarily on the first 1 to 4 days when most patients discharged were scarce. Therefore, the purpose of this study was to determine whether patients with an LOS of 2, 3, or 4 days after primary TKA and THA are at greater odds of 90-day readmission rates compared with patients with an LOS of 1 day. We hypothesize that increasing LOS after TJA will markedly contribute to the risk of postoperative readmission, regardless of comorbidities.

## Materials and Methods

### Database

A retrospective level III query was conducted from January 1, 2005, to March 31, 2014, using the Medicare Standard Analytical Files from the PearlDiver supercomputer (PearlDiver Technologies). The database contains records of more than 100 million patients and deidentified information, allowing for this study to be exempt from our institution's International Review Board approval. Patients in this study were queried using the International Classification of Diseases, ninth revision (ICD-9) coding.

### Study Groups

The study groups consisted of all patients in the database who underwent primary TKA or THA and had an LOS greater than 1 day. The database was first queried for all patients who underwent primary TKA or THA with ICD-9 procedural code 81.54 or 81.51, respectively. This query resulted in 648,758 TKA patients and 346,732 THA patients. The cohorts were filtered for patients who had an LOS of 1 day up to 4 days, using 1-day increments. Patients with an LOS of 1 day served as control subjects, whereas patients with an LOS of 2, 3, or 4 days served as the study groups. THA LOS of 1 day served as control for the THA cohort and was compared with THA study groups' LOS of 2, 3, and 4 days. TKA LOS of 1 day served as control for the TKA group and was compared with TKA study groups' LOS of 2, 3, and 4 days. In each study group, patients were matched to the control group patients according to age, sex, and Elixhauser Comorbidity Index (ECI). ECI was preferred over Charlson Comorbidity Index because ECI contains more than 30 comorbid conditions compared with Charlson Comorbidity Index, which accounts for 22 comorbid conditions. Higher ECI scores represented greater patient complexity.

### Outcomes Assessed

Ninety-day readmission rates were analyzed and compared between study groups and the control cohort. The period of ninety days was chosen for readmission rates to be compliant with the Comprehensive Care for Joint Replacement model design, stating that the episode of care begins on the day of admission and ends 90 days after discharge.^7^

### Data Analysis

Statistical analysis was conducted with the programming language R (R, University of Auckland, New Zealand) using logistic regression analysis to calculate odds ratios (OR), along with their respective 95% confidence intervals (95% CIs), and *P* values. Exact matching of patients was done based on the ECI subclass. An alpha value less than 0.05 was considered statistically significant.

## Results

### Total Knee Arthroplasty Length of Stay and 90-Day Readmissions

The results showed that patients with an LOS greater than 1 day had greater odds of 90-day readmission rates compared with those in the control group. More specifically, the data demonstrated that patients with an LOS of 2 days (OR, 2.89; 95% CI, 2.77 to 3.00; *P* < 0.001), an LOS of 3 days (OR, 2.80; 95% CI, 2.69 to 2.91; *P* < 0.001), and an LOS of 4 days (OR, 2.83; 95% CI, 2.72 to 2.95; *P* < 0.001) had greater 90-day readmission rates compared with patients who were discharged within 1 day of their hospital stay.

### Total Hip Arthroplasty Length of Stay and 90-Day Readmissions

Similar to the TKA group, patients undergoing THA with an LOS greater than 1 day had greater odds of 90-day readmission rates compared with patients with an LOS of 1 day. Specifically, the data demonstrated that patients with an LOS of 2 days (OR, 2.93; 95% CI, 2.77 to 3.10; *P* < 0.001), an LOS of 3 days (OR, 2.91; 95% CI, 2.75 to 3.07, *P* < 0.001), or an LOS of 4 days (OR, 2.91; 95% CI; 2.73 to 3.05, *P* < 0.001) after primary THA had a greater frequency of 90-day readmission rates compared with patients being discharged within 1 day of their hospital stay.

## Discussion

This study demonstrated that increased length of stay past 1 day when compared with 2, 3, or 4 days of hospital stay had an all-cause increase in 90-day readmissions. Average 90-day TJA readmission rates vary widely in the literature, ranging from 2.9% to 10.9% in THA and 3.5% to 15.6% in TKA.^1^ Infection-related and procedure-related complications, such as prosthetic dislocation or failure, seem to be the most common risk factors for readmission. Age <50 years or >80 years, body mass index (BMI) <18.5 or >30, diabetes, coronary artery disease, high American Society Anesthesiologist (ASA) score, history of bleeding disorder or cancer, perioperative transfusion, and hospital stay >5 days also represent potential risk factors.^1,2,5,6,8 9,10,11^ For instance, one such study by Saucedo et al^1^ showed that higher 90-day readmission rates were correlated with longer LOS (>5 days). Although these comorbid conditions play a possible role in readmission, another factor should take patient outcomes into consideration. This may account for satisfaction, lifestyle, and compliance, which could pose answers to these readmissions. Unplanned readmission creates a large burden for the healthcare system and is detrimental to the patient, especially when return to the operating theater is necessary.^9^ The results of this study demonstrate that greater in-hospital LOS past 1 day after primary TKA and THA increases the odds of 90-day readmission rates within the Medicare population, independent of the comorbidity index.

The study by Cram et al^4^ reported trends from 1991 to 2008 among Medicare beneficiaries who underwent primary THA (n = 1,453,493) and revision THA (n = 1,453,493), demonstrating increasing readmission rates and skilled-care discharges despite decreasing LOS over that period. It was subsequently proposed that premature discharge could pose a potential risk for unplanned readmission. However, these trends were likely affected by the fact that the number of comorbid illnesses increased almost twofold over this period. In addition, increasing discharge to skilled care likely constituted a modifiable risk factor for unplanned readmission because increased surveillance and inherent nosocomial risks could lead to greater risk of readmission. Indeed, a recent study by Bini et al^12^ showed that THA (OR = 1.9) and TKA patients (OR = 1.6) discharged to a skilled nursing facility had higher odds of 90-day readmission rates when compared with patients discharged home. Another study by Ricciardi et al evaluating a subset of 60 patients who were readmitted (matched 2:1 with 120 patients not readmitted) among 21,864 arthroplasties (TKA and THA) showed that 30-day readmission rates were higher in patients with an LOS shorter than 2 days, appearing to support the conclusions of Cram et al.^4,13^ However, this very small sampling of patients is under-representative of their total institutional population and may be subject to selection bias.

Most studies show that decreasing LOS does not seem to increase risk for readmission, both in the immediate 30-day and 90-day postoperative periods. Vorhies et al analyzed a Medicare sample from 2002 to 2007, where 30-day readmission rates remained stable for patients undergoing THA despite decreasing LOS over that period.^14,15^ Another large study from the National Surgical Quality Improvement Program (NSQIP) database looked specifically at 30-day readmission and complication rates in 31,044 TKA patients and 19,909 THA patients, finding that patients discharged from 0 to 2 days did not experience an increased risk of 30-day readmission when compared with those patients discharged 3 to 4 days after surgery.^8^ In addition, early discharge did not cause an increase in major complications in TKA and actually was shown to confer a lower risk of major complications in THA. These findings are in accordance with our study, which was similarly conducted in a large database where specific surgical data such as postoperative pain protocol, implant used, and approach type are not obtainable. However, the PearlDiver database seems to be more extensive in its ability to capture perioperative complications, including implant-specific complications, such as dislocation or periprosthetic fracture. In addition, data are available through a period of 90 days and thus may better reflect postoperative trends.^16^

Additional studies have produced comparable findings, even at the 90-day time point.^5,6,9,17^ Charpentier et al^5^ examined patients undergoing TKA (n = 46,709) in a large Michigan database and found equivalent 90-day readmission rates among patients with 1-midnight stay versus 2-midnight stays. Another much smaller institutional study by Novack et al^6^ showed equivalent 90-day readmission rates among TKA patients discharged on postoperative day 2 (POD-2) compared with a cohort with LOS ≥ 3 days, with the accelerated protocol showing no increased risk of complications, ER visits, or readmissions within 90 days. Our study supports these data and is especially relevant because it shows that increasing in-hospital stay past 1 day in a large heterogeneous Medicare population actually seems to be associated with increased risk for readmission. In addition, capturing readmission data through a longer 90-day period likely better represents the spectrum of delayed complications that can occur after TJA.

Several institutions have also implemented fast-track TJA protocols, given that there is increasing interest in evolving toward short inpatient stays and even outpatient surgery for TJA.^13,17,18,19,20^ The benefits of such protocols are not fully realized at this time, but they do likely minimize potential inpatient complications, such as nosocomial infections and venous thromboembolism, from prolonged immobilization. Raphael et al^20^ evaluated a multimodal TJA fast-track protocol where patient stay was shortened from a mean of 116 to 47 hours, describing no increase in ED visits or readmission within 30 days of discharge. A similar study by Husted et al^18^ in 1,731 TJA patients showed no increase in 90-day readmission rates or increased incidence of THA dislocation or TKA manipulation, with a mean LOS decreasing from 6.3 to 3.1 days. Sibia et al^17^ showed that 1-day LOS patients at their institution had similar 30-day and 90-day readmission rates when compared with 2-day LOS patients, were more often likely to be male, younger, with a lower BMI, and have shorter surgical times with higher incidence of spinal anesthesia. These smaller institutional studies are relevant but unlike our study do not account for variation in postoperative protocols seen in a large database.

Interestingly, a study by Otero et al^21^ examining early discharge actually showed an increased 30-day complication rate in THA patients discharged on POD-0 as compared with POD-1 but with similar readmission rates in these two groups undergoing TKA, THA, and uni-compartmental knee arthroplasty (UKA). Careful patient selection and preparation seem to play a notable role in decreasing complications in those patients who are discharged on the index day of the procedure. A recent study also focused on octogenarians, demonstrating that these elderly individuals (older than 80 years) have higher risk of 30-day readmission rates when compared with younger patients discharged on POD-1, likely because of increased nonsurgical-related readmissions.^13^ Our study specifically suggests that early discharge on POD-1 may serve to independently decrease readmission rates even regardless of the choice of perioperative protocol; however, additional investigation is needed to elucidate optimal medical protocols for early discharge in very elderly patients. Consequently, the results of this study indicate that a higher ECI may serve as an independent risk factor for developing 90-day medical complications and longer in-hospital LOS.

As a large database study, this study introduces heterogeneity by encompassing different institutional perioperative protocols, and also by controlling for comorbidity, we are able to exclude other confounding factors affecting readmission rates. However, this study is not without limitations, most of which are inherent to the use of an administrative database. The authors of this study analyzed only a single insurance provider, and it may not be an accurate representation of the effect of LOS on 90-day readmission rates. In addition, the authors used ICD-9 coding for research purposes, and it is currently postulated that there are 1.3% of coding errors within the Medicare database.^22^ There also exist the limitations regarding identifying the specific reasons for the variations in LOS beyond simply a higher comorbidity index. Procedure length, complexity, and type of intraoperative hemostasis may also influence patient LOS. Immediate postoperative complications and patient outcomes also play a factor in LOS and should be further investigated as a contributing factor to LOS. Despite these limitations, this study is vital in demonstrating the effect of overall LOS because it relates to 90-day readmission rates after primary TJA.

## Conclusion

This study supports early discharge on POD-1 for THA and TKA patients, with a lower 90-day readmission rate when compared with cohorts with a greater LOS from 2 to 4 days. These findings support efficient, timely progression to early discharge, but with the recognition that addressing patient-specific risk factors also plays a large role in preventing readmission. Fast-track protocols described in the literature will continue to be influential in lowering perioperative complications and readmission burden while also ensuring that patients are well prepared for early discharge.
